# Supplementary material for: Accumulation and control of spin waves in magnonic dielectric microresonators by a comb of ultrashort laser pulses
Source: Sci Rep. 2022 May 5;12:7369. doi: 10.1038/s41598-022-07784-2 (PMC9072547; doi:10.1038/s41598-022-07784-2)
Supplement: Supplementary file 1 — Supplementary Information. [file 41598_2022_7784_MOESM1_ESM.docx]

**Supplementary**

| 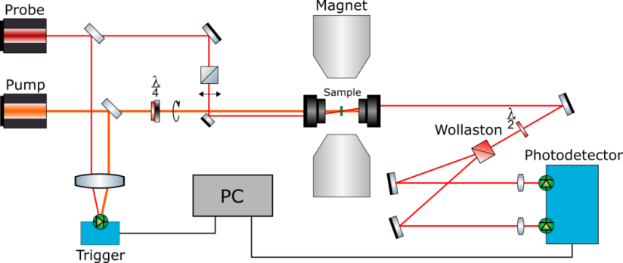 |
| --- |
| (a) |
|  |
| (b)  FIG. S1. Asynchronous optical sampling (ASOPS) method.  Asynchronous optical sampling (ASOPS) method  The magnetization precession is excited and detected using a pump-probe method based on asynchronous optical sampling (ASOPS). This method involves the operation of two independent Ti: Sapphire femtosecond lasers, which emit pump and probe pulses at the center wavelength of λ ≈ 810 nm and 780 nm. The pump pulses have a repetition rate of f_pump_ = 10 GHz, and the probe pulses have a rate of f_probe_ = f_pump_/10 ‑ Δf, where Δf = 2 kHz (Fig S1a). Thus, each probe pulse is synchronized with every 10th pump pulse with a small offset Δf. As a result, the relative time delay between the pump and the 10th probe pulse periodically increases from zero to 100 ps during a scan time of 50 μs. The fast signal is linearly stretched in time with a factor of about f_probe_/Δf and makes it accessible to fast data acquisition electronics (Fig S1b). The pump and probe beams are focused on the sample using a single reflective microscope objective with a magnification of 15, including 4 sectors through which light can enter and exit, pump beam diameter 9 μm, probe beam diameter 7 μm . The second objective of the microscope is used to collect and collimate the probe beam in transmission geometry. Stray light from pump laser in the detection path was reduced by using interference bandpass filter centered at the probe wavelength. |

| 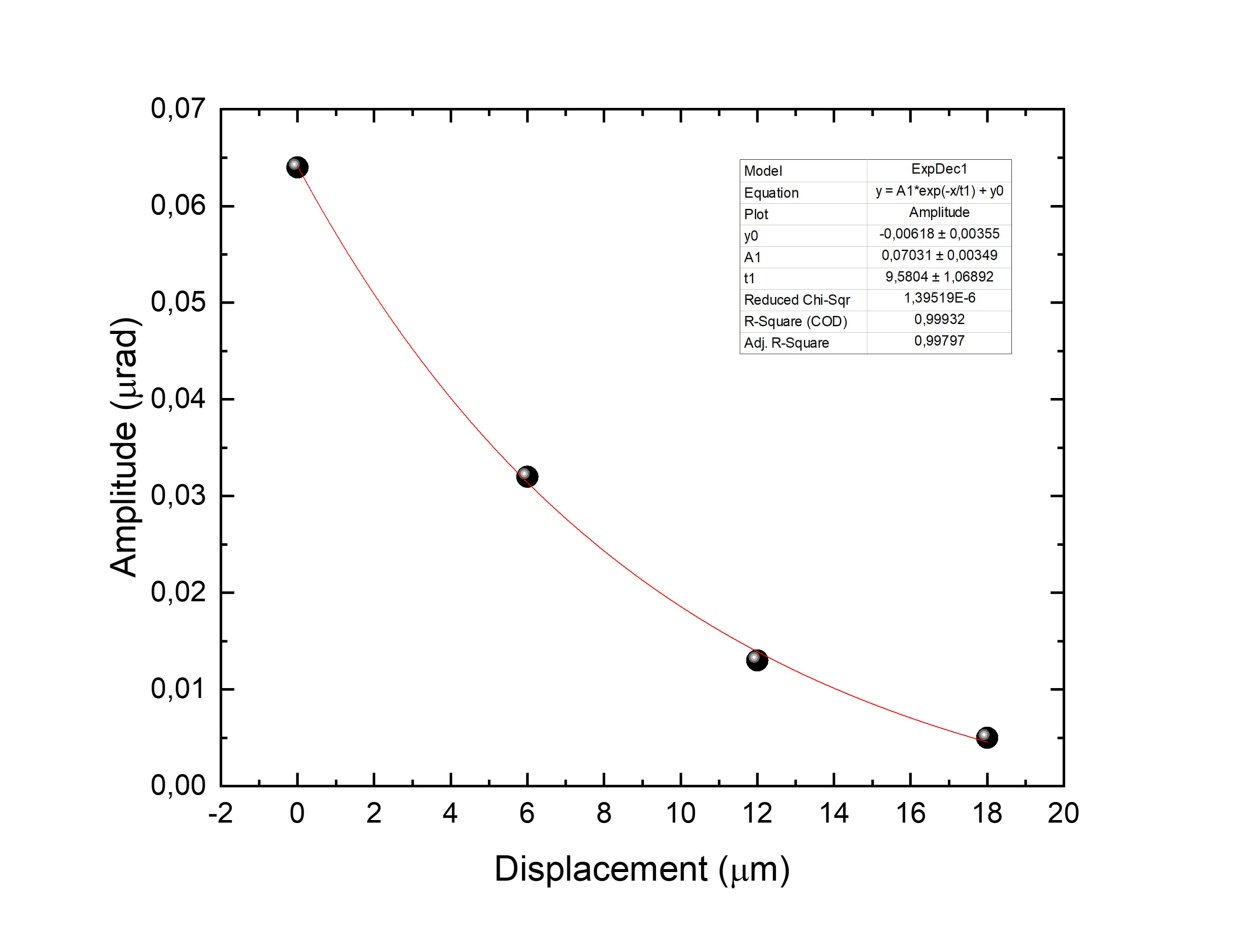 |
| --- |
| FIG. S2 Exponential decay of spin waves as the sample beam moves away from the pump beam. The black circles indicate the experimental data on the amplitude of the spin waves. The red line is the fit curve. The external magnetic field for this experiment was 2.97 kOe. The pump fluence is 5,2 µJ/$\mathrm{cm}^{2}$ |
